# A Scalable Service to Improve Health Care Quality Through Precision Audit and Feedback: Proposal for a Randomized Controlled Trial

**DOI:** 10.2196/34990

**Published:** 2022-05-10

**Authors:** Zach Landis-Lewis, Allen Flynn, Allison Janda, Nirav Shah

**Affiliations:** 1 Department of Learning Health Sciences University of Michigan Medical School Ann Arbor, MI United States; 2 School of Information University of Michigan Ann Arbor, MI United States; 3 Department of Anesthesiology University of Michigan Medical School Ann Arbor, MI United States

**Keywords:** learning health system, audit and feedback, anesthesiology, knowledge-based system, human-centered design

## Abstract

**Background:**

Health care delivery organizations lack evidence-based strategies for using quality measurement data to improve performance. Audit and feedback (A&F), the delivery of clinical performance summaries to providers, demonstrates the potential for large effects on clinical practice but is currently implemented as a blunt *one size fits most* intervention. Each provider in a care setting typically receives a performance summary of identical metrics in a common format despite the growing recognition that *precisionizing* interventions hold significant promise in improving their impact. A precision approach to A&F prioritizes the display of information in a single metric that, for each recipient, carries the highest value for performance improvement, such as when the metric’s level drops below a peer benchmark or minimum standard for the first time, thereby revealing an actionable performance gap. Furthermore, precision A&F uses an optimal message format (including framing and visual displays) based on what is known about the recipient and the intended gist meaning being communicated to improve message interpretation while reducing the cognitive processing burden. Well-established psychological principles, frameworks, and theories form a feedback intervention knowledge base to achieve precision A&F. From an informatics perspective, precision A&F requires a knowledge-based system that enables mass customization by representing knowledge configurable at the group and individual levels.

**Objective:**

This study aims to implement and evaluate a demonstration system for precision A&F in anesthesia care and to assess the effect of precision feedback emails on care quality and outcomes in a national quality improvement consortium.

**Methods:**

We propose to achieve our aims by conducting 3 studies: a requirements analysis and preferences elicitation study using human-centered design and conjoint analysis methods, a software service development and implementation study, and a cluster randomized controlled trial of a precision A&F service with a concurrent process evaluation. This study will be conducted with the Multicenter Perioperative Outcomes Group, a national anesthesia quality improvement consortium with >60 member hospitals in >20 US states. This study will extend the Multicenter Perioperative Outcomes Group quality improvement infrastructure by using existing data and performance measurement processes.

**Results:**

The proposal was funded in September 2021 with a 4-year timeline. Data collection for Aim 1 began in March 2022. We plan for a 24-month trial timeline, with the intervention period of the trial beginning in March 2024.

**Conclusions:**

The proposed aims will collectively demonstrate a precision feedback service developed using an open-source technical infrastructure for computable knowledge management. By implementing and evaluating a demonstration system for precision feedback, we create the potential to observe the conditions under which feedback interventions are effective.

**International Registered Report Identifier (IRRID):**

PRR1-10.2196/34990

## Introduction

### Background

There is nearly universal agreement regarding the need to improve care quality and health outcomes. All health care delivery organizations measure care quality and outcomes, increasingly via electronic clinical quality measures [1] and dashboards [2,3]. However, these organizations lack evidence-based communication strategies for implementing quality measurements to work to improve their performance [4,5]. The most common approach is audit and feedback (A&F), the delivery of clinical performance summaries to providers, which demonstrates the potential for large effects on clinical practice [6-8]. However, A&F too often produces negligible effects [5,9], creating little more than distraction for providers who are fatigued by information chaos [9-11].

As currently implemented, A&F is a blunt *one size fits most* intervention. Each provider in a care setting typically receives identical metrics in a common format despite the growing recognition that *precisionizing* interventions hold significant promise in improving their impact [12-15]. A precision approach to A&F prioritizes *display of information for the single metric* that, for each recipient, carries the highest value for performance improvement, such as when the metric’s level drops below a benchmark or standard for the first time, revealing an actionable performance gap [16-19]. Furthermore, precision A&F would use an optimal message format (including framing and visual displays [20-24]), based on what is known about the recipient and their context, to improve message interpretation while reducing the recipient’s cognitive burden [25-28]. Well-established psychological principles, frameworks, and theories form a feedback intervention knowledge base to achieve precision A&F [16-19,29-33].

From an informatics perspective, precision A&F requires a knowledge-based system that enables mass customization by representing knowledge that is configurable at the group and individual levels. A precision A&F service uses this knowledge as *requirements* (necessary characteristics for message acceptability) and *preferences* (the relative importance of message characteristics to the recipient) to generate messages that are more likely than a *one size fits most* report to positively influence clinical decision-making and practice. An equally important informatics challenge is enabling widespread improvement through a service for precision A&F at scale. A *scalable precision A&F service* must function as an infrastructure compatible with a wide range of computing environments and supporting a wide range of clinical domains.

We developed and tested a prototype knowledge-based system for precision A&F in anesthesia care. Preliminary data show that provider preferences are not uniform, suggesting that a platform for computable knowledge is necessary to support scalable precision A&F. The Knowledge Grid platform, developed at the University of Michigan, has been shown to support *precisionizing* for clinical decision support (CDS) systems [34-36]. On the basis of our prior work, the proposed project will advance the creation of more general services for precision A&F by applying the service in anesthesia care as a demonstration domain.

### Objectives

Three aims will direct this research. Our first aim is to systematically capture recipient requirements and preferences for precision A&F messages. We will identify requirements via human-centered design [37] with a provider sample from a national anesthesia quality improvement consortium of >50 hospitals and >5000 providers who receive a monthly *one size fits most* A&F email [38]. A web-based survey will elicit individual provider A&F email preferences through pairwise comparison [39]. A cluster analysis [40] of preference data will be used to identify group preferences. Our guiding research question for this aim is as follows: What differences exist in the requirements and preferences for A&F messages in anesthesia care?

Our second aim is to implement and assess a demonstration service for scalable precision A&F. We will enhance the interoperability of our system by adopting Knowledge Grid’s scalable and extensible approach based on digital knowledge objects [41] and common web service application programming interface (API) technology. We will integrate our service to add an individualized message to the existing *one size fits most* A&F email sent monthly to >5000 providers. We will evaluate the performance of the precision A&F service using existing quality measurement data from >50 hospitals and conduct usability testing [42,43] with a diverse sample of providers and hospitals.

Our third aim is to assess the effects of a precision A&F service on care quality and intervention engagement. We will conduct an embedded, pragmatic cluster randomized trial of precision A&F–enhanced email versus a standard *one size fits most* A&F email to anesthesia providers. We hypothesize that providers receiving precision A&F will increase (1) care quality for improvable measures and (2) email engagement (click-through and dashboard login rates) when compared with providers receiving standard A&F emails. We will assess unintended consequences in a mixed methods process evaluation [44,45].

We aim to demonstrate the mass customization of A&F to improve care quality at a large scale. Following the National Institutes of Health (NIH) National Library of Medicine’s vision of data *to knowledge* using a *digital objects approach* for computable knowledge, we will create potential for system-level learning about A&F to improve care quality.

## Methods

### Overview

**Figure 1:**
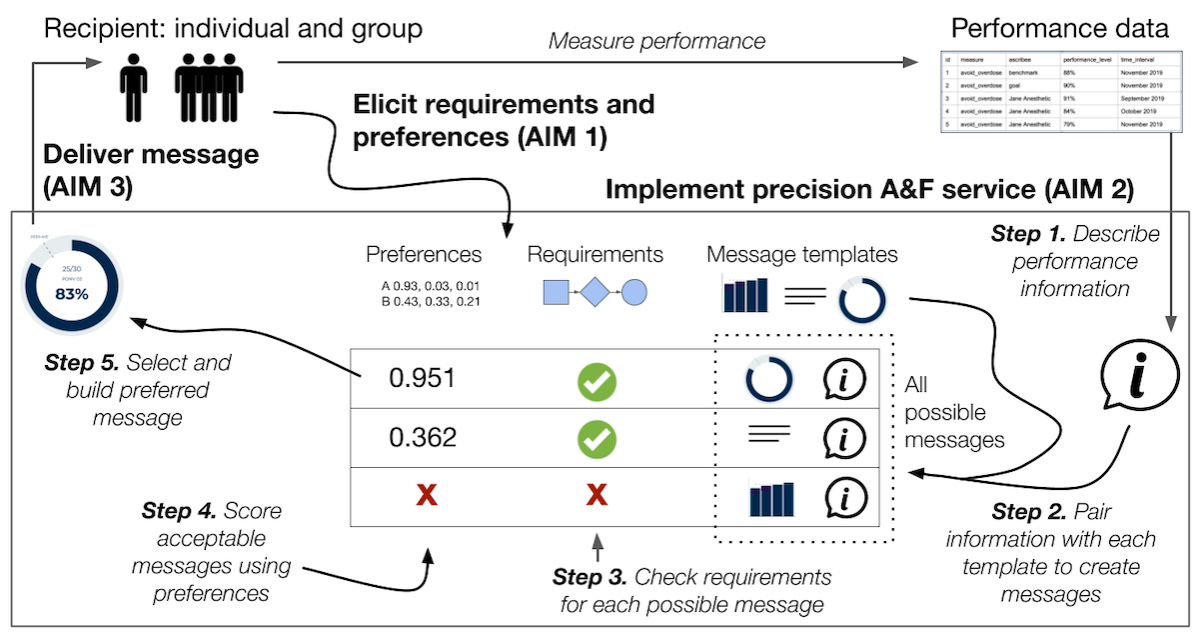
A precision feedback service. A&F: audit and feedback.

We propose to achieve our aims by conducting 3 studies (Figure 1). Aim 1 is a requirements analysis and preferences elicitation study. Aim 2 is a software service development and implementation study. Aim 3 is a cluster randomized controlled trial of a precision A&F service with a concurrent process evaluation.

### Ethics Approval

The proposed studies were approved by the University of Michigan Medical School Institutional Review Board as an umbrella project (IRBMED #HUM00194224) and for Aim 1 studies as exempt (IRBMED #HUM00204206).

**Figure 2:**
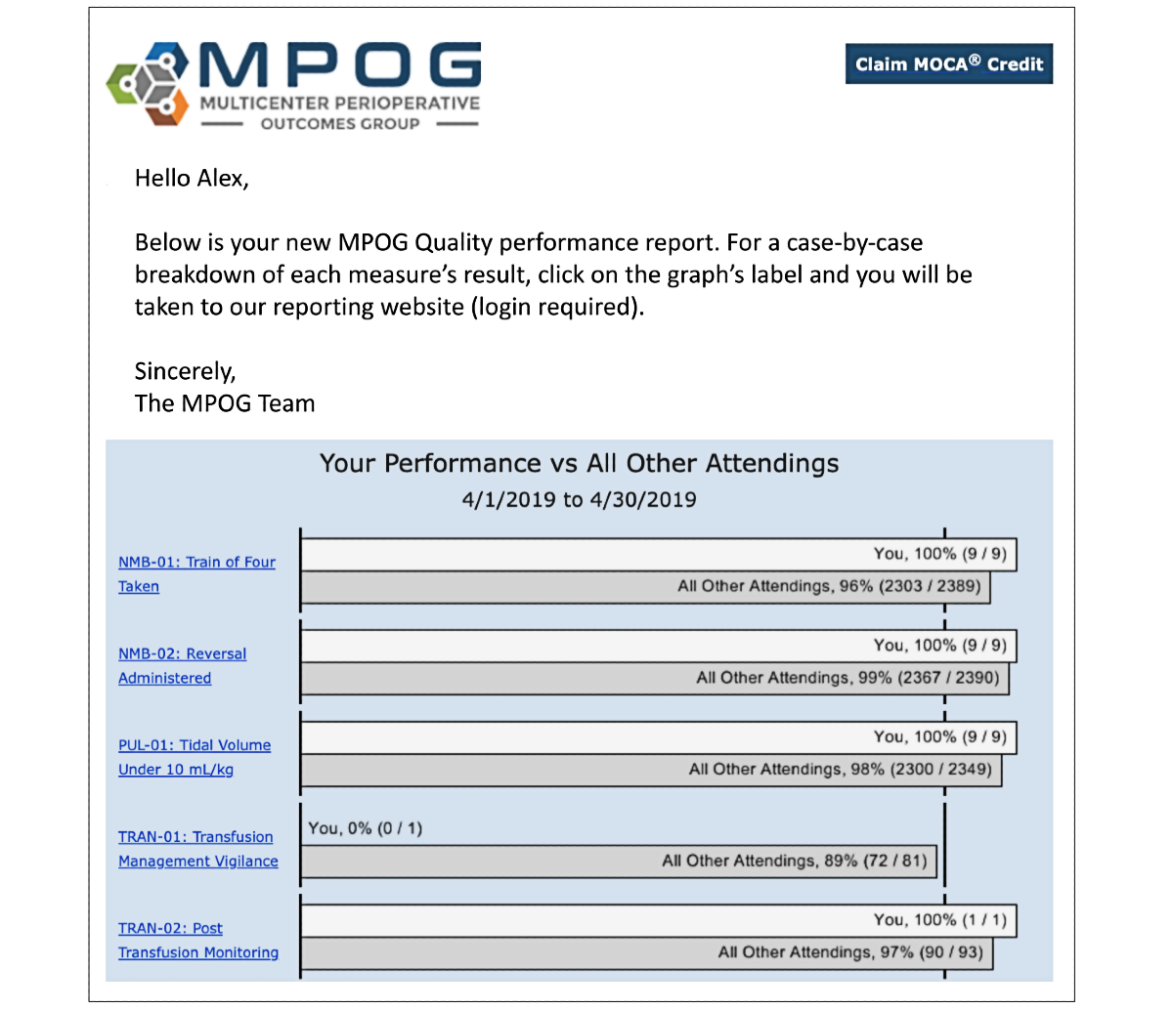
An example provider feedback email from the Multicenter Perioperative Outcomes Group (MPOG) setting.

This study will be conducted with the Multicenter Perioperative Outcomes Group (MPOG), a national anesthesia quality improvement consortium with >60 member hospitals in >20 states [38]. MPOG is housed at the University of Michigan and maintains a quality improvement infrastructure that represents a large-scale platform for research in precision feedback, reaching approximately 6000 anesthesia providers in monthly A&F emails. MPOG providers include certified registered nurse anesthetists, anesthesiologist attendings, and resident physicians. MPOG provider feedback emails are delivered with approximately 4 to 20 quality measures per provider, assessed, and attributed to the individual provider’s care quality and clinical outcomes each month. Measures are presented either as the rate of operative case success (for process measures) or as the rate of flagged or failed cases (for outcome measures, also called inverse measures), using criteria developed and maintained for quality improvement use in the MPOG consortium [46]. Currently, MPOG sends these data in a monthly automated (*standard*) A&F email that displays all measures attributed to the recipient in a bar chart, with each measure showing bars comparing provider performance to the MPOG average for that measure (Figure 2). Process measures have a 90% goal, and outcome (inverse) measures have lower, measure-specific goals, against which the provider and their institutional peer average performance can be compared. The email directs recipients to a clinical quality dashboard also maintained by MPOG, within which providers can review their patients’ case-level data to identify opportunities for improvement.

### Aim 1: Systematically Capture Recipient Requirements and Preferences for Precision A&F Messages

At a large scale, the usability of digital interventions becomes critical for their success [47]. Usability of precision A&F requires the elicitation of a sample of recipients’ requirements and preferences at the group and individual levels. We propose to achieve usable precision feedback interventions with complementary customization strategies (Table 1).

**Figure 3:**
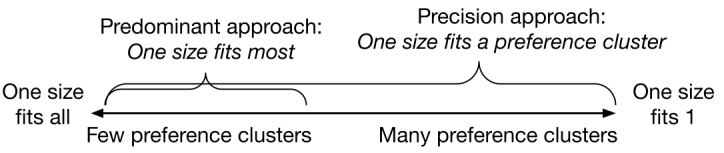
A one size fits *n* spectrum.

We use a novel approach comprising three customization strategies simultaneously, based on knowledge availability: (1) theory-based customization using the characteristics of an individual’s performance data, (2) group-level segmentation and targeting based on requirements and preference clusters (Figure 3) obtained via human-centered design activities and cluster analysis of preference data, and (3) full tailoring using individual-level requirements and preferences obtained through user configuration of requirements and participation in a conjoint analysis survey (Table 1). This approach enables a precision A&F service to provide communication that is robust to missing knowledge at the individual or group level.

We will identify requirements via human-centered design methods [37,48] with a sample of providers who participate in the MPOG monthly provider email feedback program, receiving a standard, *one size fits most* A&F email [38]. A web-based survey will be used to elicit individual provider A&F email preferences via a pairwise comparison approach [39]. A cluster analysis [49] of individual preference data will be used to identify group preferences (Table 1).

**Table 1:** Precision audit and feedback knowledge for intervention success.

Knowledge class and intervention knowledge		Causal pathway component	Knowledge acquisition method	Customization strategy	Precedence
**Theory**					
	Requirements	Preconditions	Representation of psychological theories and frameworks	Theory-driven customization	Low
	Preferences	Moderators	Representation of psychological theories and frameworks	Theory-driven customization	Low
**Group**					
	Requirements	Preconditions	Human-centered design	Targeting and segmentation	Medium
	Preferences	Moderators	Cluster analysis of conjoint analysis data	Targeting and segmentation	Medium
**Individual**					
	Requirements	Preconditions	Provider configuration of settings	Tailoring and individualization	High
	Preferences	Moderators	Conjoint analysis survey	Tailoring and individualization	High

**Figure 4:**
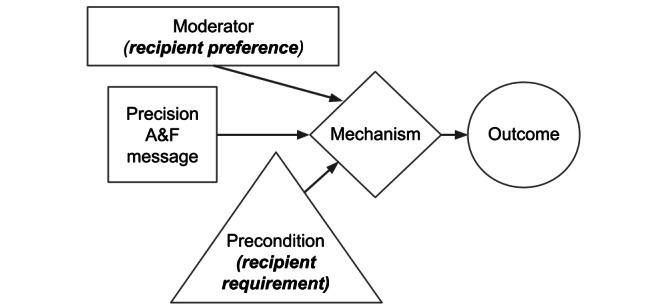
A causal pathway model for precision audit and feedback (A&F) interventions.

Our guiding research question for this aim is as follows: “What differences exist in provider requirements and preferences for A&F messages in anesthesia care?” We will identify and describe these differences in terms of the message information and format [50], based on 2 application ontologies we developed for this purpose using Basic Formal Ontology [51] as an upper-level ontology. The Performance Summary Display Ontology (PSDO) [52] describes information and formatting elements of performance summaries. In a preliminary evaluation of PSDO, we successfully described published examples of feedback reports and dashboards from a wide range of clinical settings [50]. The Causal Pathway Ontology describes influence pathway components, including mechanisms, preconditions, moderators, and outcomes (Figure 4), based on a causal pathway modeling approach [53].

When used together, PSDO and the Causal Pathway Ontology provide a well-defined domain for reasoning about performance summaries within feedback messages and their anticipated effects.

**Figure 5:**
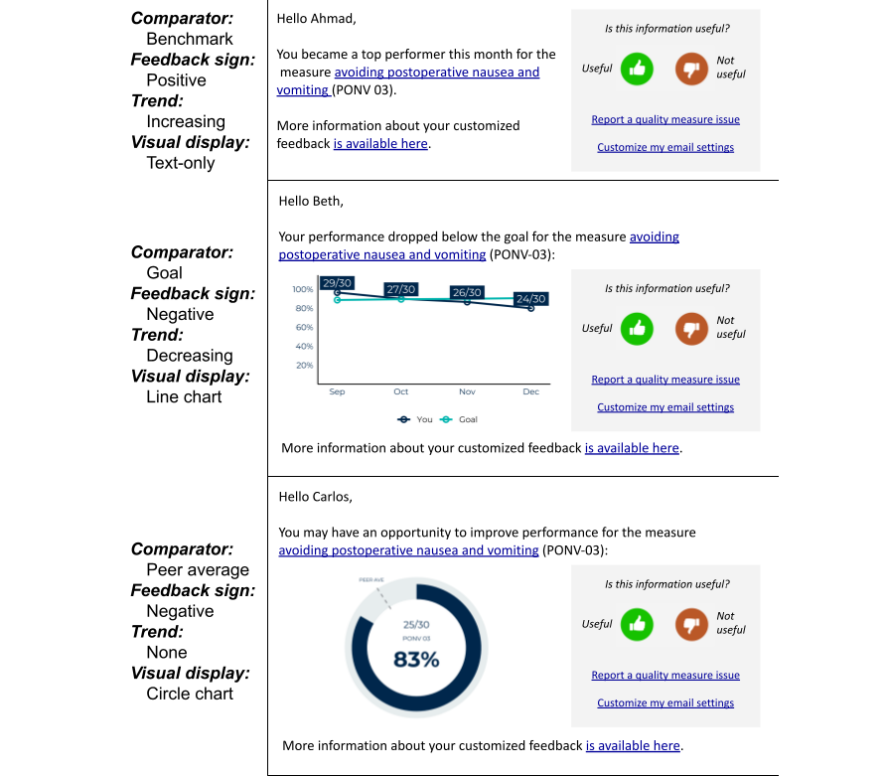
Prototype precision feedback email messages.

To develop group-level requirements, we will interview a sample of approximately 50 providers from up to 25 MPOG member hospitals to collect qualitative data on precision feedback requirements. We will ask participants to *think aloud* while they read prototype precision feedback messages to observe their cognitive processing of the precision feedback prototypes (Figure 5). We will analyze the interview data using template analysis, in which 2 researchers will use a codebook we developed for this purpose. New themes will be developed as requirements in the form of user stories during the analysis phase [37]. After coding is complete, requirements will be coded with classes from our ontologies and from the Behavior Change Intervention Ontology [54] to develop computable user stories at the group level.

To elicit preferences, we will conduct a web-based survey using a pairwise comparison method with an adaptive conjoint analysis. Adaptive conjoint analysis is a marketing research method [55,56] increasingly used to elicit patient preferences in health care [57] and has been used to identify *preference phenotypes* [58,59]. Preferences can be represented quantitatively as utilities that indicate the relative importance of specific attributes and levels (ie, part-worth utilities) of a product or service. These preference models can be used to represent the relative importance of message characteristics specified in PSDO based on their role as preconditions for feedback intervention success. We developed a web-based survey using 1000Minds software (1000Minds Ltd) [60], which presents participants with pairwise comparisons of message characteristics in 3 dimensions of preconditions (comparator, feedback sign, and trend) and 1 dimension for the visual display type. We will recruit MPOG providers from diverse hospitals and geographic regions, and with diverse demographics, to participate in the study and to complete the 10-minute survey. We estimate that recruiting approximately 300 participants is feasible based on a 10% response rate observed in our previous recruitment in this population.

The web-based survey data will be used to produce individual-level preference models via an adaptive conjoint analysis of provider preferences for precision feedback emails. 1000Minds uses a method called Potentially All Pairwise RanKings of all possible Alternatives that allows reduction of the total number of comparisons to be made through assumed transitivity of preference and that permits participants to indicate indifference toward the 2 choices to be compared [39]. This approach also uses repeated questions to check the consistency of preferences as a test of the assumption of transitivity for each participant. This analysis will produce part-worth utilities, also called preference weights, that can be averaged across all participants to establish group mean weights.

Individual rankings for all attributes from the adaptive conjoint analysis can be used for a cluster analysis to identify groups of participants with similarities across one or more preference characteristics. We will conduct a hierarchical cluster analysis [40] of preference data using participant characteristics to identify group-level preferences of population segments for the targeting and segmentation strategy of the precision A&F service (Table 1). We will use the NBClust [61] package in R (R Foundation for Statistical Computing) [62] to identify the optimal number of clusters for our data.

There are potential problems that may require alternative approaches to enable our successful completion of this aim. Self-selection bias in recruitment could reduce participant representativeness of the provider population. To prevent this issue, we will actively recruit providers who do not hold the position of the MPOG QI Champion and who do not routinely use the dashboard, which we estimate to be a large proportion of the population. On the basis of our preliminary studies, we anticipate a significant variation in preferences. In the unlikely event that preferences for feedback emails are highly similar across all dimensions of all message characteristics (including comparator, feedback sign, trend presence, and visual display), the diversity of providers’ individual performance levels will nevertheless enable precision A&F messages to be individually prioritized using theoretical requirements and preferences.

Using utilities to represent provider preferences imposes several assumptions that may not hold: provider preferences may not be complete, may not be linear in probability, and may not be stable over time. The collected data will enable us to learn about the validity of these assumptions. We will test for consistency and stability of preferences by conducting 2 rounds of the adaptive conjoint analysis (years 1 and 3) to observe preference changes. We will also consider (1) diversity of participants along demographics and professional roles, (2) representativeness of the provider population, and (3) diversity of organizations and clinical settings (community hospitals and academic medical centers) from which participants are recruited, and strive to maximize these and other forms of diversity and representativeness. We will strive to recruit participants from a representative gender mix within the anesthesia provider population.

### Aim 2: Implement and Assess a Demonstration Service for Scalable Precision A&F

We will make our knowledge-based system interoperable by conforming to open standards in a scalable and extensible service model. We will do this by developing a small collection of modular digital knowledge objects [41] that can each be shared and managed independently and a corresponding modularized web service API approach. Next, we will move from a *one size fits most* A&F email message sent to >6000 anesthesia providers each month to test the processing of data for mass-customizing message content computed by and coming from the precision A&F service. We will then test service performance in terms of data processing capability using existing quality measurement data from a subset of the available 60 hospitals and conduct usability testing [42,43] to assess feasibility. Our research goal for this aim is for the service to become operational and pass performance benchmarks for system functioning at a national scale, processing data for at least 30 hospitals in at least two separate regions of the United States, but not yet sending precision feedback messages at this step.

Precision A&F may have a large impact when it can be easily deployed and managed as a scalable A&F web service by quality improvement organizations that serve many providers. Demonstrating success at a large scale requires a technical platform that enables mass customization of computable knowledge capabilities provided by the Knowledge Grid platform. This system development and implementation study is consistent with the NIH National Library of Medicine’s vision of a computable knowledge approach using digital objects that can be maintained and curated in accordance with the FAIR (Finable, Accessible, Interoperable, and Reusable) principles [63].

We will package our precision A&F specifications and algorithms in digital knowledge objects for each type of recipient knowledge (Table 1) and for the major components of the precision feedback system (Figure 1). This packaging step is to establish the easily deployable and shareable precision A&F service, built on Knowledge Grid technology and processes, including the following: packaging and deploying knowledge objects, standing up knowledge object–backed service APIs using OpenAPI standard web service specifications, creating a deployment specification to facilitate deployment into existing information technology environments, conducting precision A&F web service testing, including unit and integration testing, and finally implementing the ready-to-use service.

The implemented precision A&F service will routinely and automatically apply requirement and preference knowledge about recipients in a just-in-time approach based on a specified order of application and precedence (Table 1). Given that many recipients are not expected to provide individual-level requirements or preferences, we will at a minimum use theory-based requirements knowledge for precision A&F for all recipients. Before each monthly feedback cycle, we will reapply new requirements or preference knowledge as part of an automated and routine system adaptation process for all participants. Group requirements and group mean preferences will be automatically assigned based on computable user stories and weighted means, respectively, for the recipients’ professional role and site data. Cluster-based preferences will be automatically assigned based on the detection of a significant statistical association (eg, using multinomial logistic regression) between recipient characteristics and a preference cluster. Individual recipients will be routinely offered the opportunity to provide requirements via a precision A&F dashboard–based configuration page and to provide preference data via the web-based survey developed in Aim 1. Individual requirements and preferences will take the highest precedence, and will be used to overwrite any automatically assigned group or theory-based preferences (Table 1).

To test the function of the system, we will generate synthetic requirements and preference data and collect existing MPOG performance data for analysis. We will test the performance of the service for the processing of email-based precision A&F for approximately 6000 anesthesia providers but will not yet send any messages generated at this step. We will optimize system functions to minimize production time and computation costs within a monthly reporting cycle.

We will implement the service within MPOG’s provider email program, such that providers at any institution selected for piloting can receive precision A&F messages for testing purposes. We will recruit a sample of up to 50 providers from 4 institutions, including 2 community hospitals and 2 academic medical centers. We will invite participants to use the web-based survey to generate individual preference data and to configure their individual precision A&F email requirements. We will conduct *live usability testing* [43] by scheduling video calls to conduct a think-aloud testing of a sample of emails received with providers’ current performance information. We will assess the collection of email engagement data using click-through data for email links.

As the Knowledge Grid technology and the common standards it uses have already been demonstrated to function as needed for our purposes, we do not anticipate significant technical barriers to achieving this aim. A possible problem is unanticipated complexity resulting from diverse requirements, organizational culture, and ecosystem changes, as requirements are specified for precision feedback. To address this problem, if significant, we will reduce the scope of the demonstration system in terms of the number of performance measures to be maintained and will implement the system within a reduced number of participating hospitals that have a larger proportion of providers before expansion throughout the consortium. This aim will be successfully completed when the precision A&F service becomes operational at its sites of implementation using the Knowledge Grid platform technology and can pass performance benchmarks for system functioning at a national scale, processing data for at least 30 hospitals in at least two separate regions of the United States.

We will use the following software development strategies to ensure a robust and unbiased approach: (1) use open standards that are broadly adopted for knowledge representation, software development, and metadata management and (2) develop open-source software in a public repository (GitHub) from the start (open development process) under an open-source license. Throughout this process, we will also ensure a robust and unbiased approach by eliciting our values as a project team and reviewing the organization’s values to seek agreement on our fundamental goals. Furthermore, we will consider the diversity of our team members to strive to reduce bias through diversity and inclusion practices, such as sending position openings to communities and organizations with team members who may be underrepresented in our team and department. Furthermore, we will consider the diversity of participants, including gender, in interviews and seek opportunities to involve participants in decision-making for the design of the system.

### Aim 3: Assess the Effects of a Precision A&F Service on Care Quality and Email Engagement

**Figure 6:**
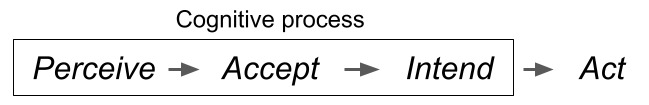
A process model for feedback intervention success.

Behavior change theories offer many potential explanations of what works when using feedback interventions to influence human behavior [64-67], and the formalization of these theories is ongoing [68]. Successive efforts have aligned key theories [29,30,69] around a sequence of cognitive steps that occurs between the perception of feedback and action taken in response, resulting in a common sequence of constructs that represent necessary steps for feedback intervention success [17-19,29,30] (Figure 6).

**Figure 7:**
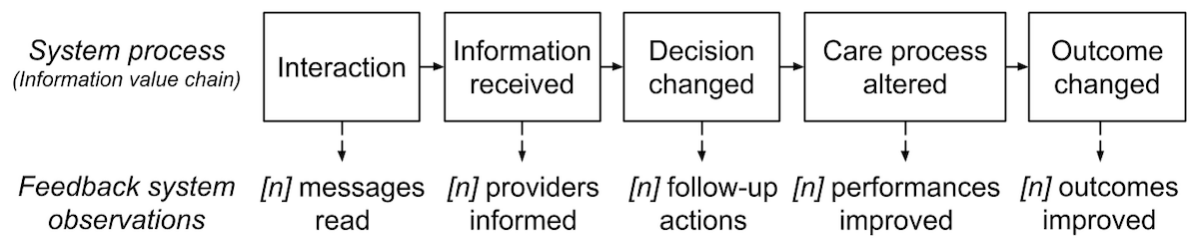
An information value chain for feedback intervention success.

Information value chain theory [70], a theory developed for the purpose of assessing technology success, presents a strikingly similar sequence of steps and has been applied to A&F to support recognition of several points of failure along an information value chain [44,45,70]. For example, when feedback is delivered via email, not all providers will open and read every email. Of those who read the email, not all decide to follow up on the information (Figure 7). A value chain analysis can reveal how a high proportion of information in A&F reports may lack value from the outset and reduce the likelihood of ongoing provider engagement with the intervention. When each step in the chain can be associated with an observable event via email link tracking and log file analysis, an information value chain approach offers an unobtrusive, theory-based evaluation approach for A&F [71] with high potential to reveal how feedback interventions are made more effective.

We will conduct an embedded cluster randomized trial of precision A&F–enhanced email versus a standard A&F email to anesthesia providers. We hypothesize that providers receiving precision A&F will increase (1) care quality for improvable measures and (2) email engagement (click-through and dashboard login rates) when compared with providers receiving standard A&F emails. We will also assess unintended consequences in a mixed methods process evaluation [44,45].

The selected outcomes for the trial are consistent with information value chain theory [45], reflecting the influence of information received on decision-making and behavioral response in terms of observable actions. For example, an observable response action indicating *decision changed* success is clicking on a link inside the enhanced or standard A&F email; performance on improvable process measures reflects *care process altered* success and performance on outcome measures reflects *outcome changed* success. Email engagement rates are used to support the retrospective calculation of event probabilities that can be associated with the characteristics of the recipient, group, and email message.

To understand the potential impact of precision A&F, we will implement and study the effects of a demonstration system at a large scale, supporting the implementation and maintenance of computable knowledge about feedback recipients’ requirements and preferences in a wide range of settings. As there is a potential for software-associated unintended adverse consequences [11], we will conduct a mixed methods process evaluation that is concurrent with the trial to inform our understanding of the observed effects.

Our study design includes a 2-arm cluster randomized controlled trial and a mixed methods process evaluation. In this study, the intervention arm will receive an enhanced monthly email containing precision A&F, and the control arm will receive the standard *one size fits most* A&F monthly email. Secular trends and regression to the mean effects, due to selection of measures on which performance is relatively low will be balanced across the 2 arms. All data for the trial will be provided by MPOG via its quality measurement data collection and analysis infrastructure, which is housed at the University of Michigan. The primary outcome will be the average postintervention performance level (*P*) for improvable measures (subscript *m*), where

P_m_ = 100 × (number of operative case successes)_m_ / (total number of operative cases)_m_
**(1)**

Each participant’s performance level on each measure will thus vary from 0 to 100. Scores on some MPOG measures are historically and consistently high, creating the potential for ceiling effects. The improvable measures are defined as clinical process measures with a mean score historically <98% for all providers participating in the MPOG provider feedback email program. For the proposed study, we will determine the set of improvable measures using up-to-date performance data. On the basis of the current performance data from all MPOG providers, 7 improvable measures may be included. The following are three examples:

- BP-02: Avoiding Monitoring Gaps. Percentage of cases where gaps >10 minutes in blood pressure monitoring are avoided.
- NMB-01: Train of Four Taken. Percentage of cases with a documented Train of Four after the last dose of a nondepolarizing neuromuscular blocker.
- PUL-02: Protective Tidal Volume, 8 mL/kg predicted body weight. Percentage of cases with median tidal volumes ≤8 mL/kg.

Secondary outcomes will be average rates of email engagement in the postintervention period, including email click-through rate (CTR) and dashboard login rate (L), where

CTR = 100 × (number of recipient’s emails with a clicked link) / (total number of emails) **(2)**

L= 100 × (number of months with an email recipient’s dashboard login event) / (total number of months) **(3)**

Each participant’s CTR and dashboard login rate will vary from 0 to 100. Email CTR is an essential measure for advertising systems that is widely used in email marketing studies. CTR will be measured using link tracking with unique URLs for each email link in the precision A&F and standard A&F emails. Dashboard logins will be measured using log file analysis. The MPOG-wide dashboard login rate is estimated to be low, with approximately 6% of MPOG providers logging in each month.

Predictor variables will include discrete and continuous measures. Discrete variables will include the recipient’s (1) study arm (*precision-enhanced messages, standard messages),* (2) hospital type *(community, academic medical center)*, and (3) professional role (*certified registered nurse anesthetist, resident, attending*). Continuous variables will include (1) average preintervention performance level (for improvable measures, calculated in the same way as postintervention performance) and (2) total number of months since the first month of participation in MPOG.

MPOG has >60 hospitals and a population of >6000 providers; however, because of the potential for hospital-level factors limiting participation, such as electronic health record implementation or reorganization activities, we estimate that at least 30 hospitals will be included. We will exclude providers who (1) end participation in the MPOG provider feedback email program for any reason, (2) change institutions, or (3) change professional roles (eg, transition from resident to attending) before the end of the intervention period. After exclusion of individual providers, we anticipate that we will engage approximately 3500 providers.

We will collect 1 year of retrospective performance and email engagement data at participating hospitals during the preintervention period. We will randomize hospitals to be in either arm of the study by using a restricted randomization approach to minimize baseline imbalance [72]. All providers participating in the MPOG email program at intervention sites will begin receiving precision A&F–enhanced emails at the start of the intervention period. Providers in the control sites will continue to receive the standard monthly A&F email. All providers at all participating hospitals will be notified of the study and offered the option not to participate. We expect few *opt-outs* as all providers in the sample are established MPOG participants, and the study will not intrude on their time.

To analyze the primary and secondary outcomes, we will use two-level (hospital and individual) hierarchical linear modeling. We will account for clustering and report the intraclass correlation coefficients. In this statistical model, the primary hypothesis for this study is explored through a main effect by study arm (precision-enhanced messages vs standard messages). This study has a large population of providers (estimated 1750 per study arm) across 30 hospitals. In exploring our primary hypothesis, we will test for differences in improvable measure performance across the 2 treatment groups at the individual provider level. The sample size will allow us to detect small differences across the study arms. On the basis of the most recent available MPOG data, the SD of performance at the individual level, averaged across 7 improvable performance measures, was 23 scale points. From this, we determined that our sample size has 80% power at 2-tailed α=.05 to detect a difference of 2.2 scale points across the 2 study arms (Cohen *d*=0.096).

We will conduct a process evaluation to understand the context, implementation process, and mechanisms associated with the precision A&F service during the trial period. We will conduct quantitative and qualitative methods in alignment with a process evaluation framework for complex interventions [73]. In the quantitative evaluation, we will monitor events in the information value chain and calculate event probabilities for email open rates, follow-up decisions, and performance improvements in processes and outcomes. We will analyze relationships between message characteristics and event likelihood and calculate the expected utility of messages using utility and likelihood. We will analyze feedback from email message usability questionnaires and responses to answer the following questions:

1. What proportion of each step in the information value chain was achieved?
2. What information was correlated with higher completion of steps in the information value chain?
3. What message formatting was correlated with higher completion of steps in the value chain?
4. What mechanisms, preconditions, and moderators were correlated with higher completion of the information value chain?

We will conduct a qualitative process evaluation to understand perceptions of the precision feedback and unanticipated adverse effects of the intervention. We will conduct qualitative phone or video call interviews with stakeholders and providers from 3 to 5 sites. We will thematically analyze the effects of the intervention using the Tailored Implementation of Chronic Disease framework in a template-editing approach. We will also aim to identify the mechanisms of action reported by participants. The qualitative process evaluation will aim to answer the following questions:

1. What potential differences in the intervention effect may be due to sex or gender and race or ethnicity?
2. What theoretical mechanisms of action appear to have been used for precision A&F emails?
3. To what extent might unintended adverse consequences of precision A&F have occurred?
4. What differences exist in provider receptiveness to precision feedback emails, in association with recipient, group, or message characteristics?

As a digital intervention, standardization for study participation and adherence to the study protocol are mostly infrastructural issues that have been addressed by MPOG in its establishment of A&F. We expect that the automation of feedback delivery will therefore benefit from a high level of standardization and adherence. However, we also will use our secondary outcomes of email engagement to support quality assurance. We will develop measures for the quality of the intervention and monitor their performance to identify software-based issues with the planned delivery of precision feedback.

For the trial, all data collected are part of the routine monthly data collected by the MPOG consortium, which has established a mature infrastructure that includes standardization, monitoring, and quality assurance processes. We will extend the existing MPOG infrastructure to conduct the trial, leveraging an extensive body of existing resources and infrastructure for routine A&F. Data management and quality control for the study will be managed by the MPOG team that routinely manages and analyzes performance data. We will develop software-based statistical analysis for quality control of the study data to estimate the effect of the interventions and to support monitoring of the intervention effect before the end of the trial. We have planned for a 6-month period to allow for adequate time to complete data analysis following the trial. We anticipate that the quantitative analysis can be completed within weeks of the conclusion of the trial and that the bulk of the 6-month period will allow time to complete a qualitative process evaluation of the trial, which will be ongoing and concurrent with the randomized controlled trial.

A foreseeable problem for the trial is that performance is high overall, which reduces the potential impact of precision feedback on performance. Our process evaluation will enable us to observe reactions to positive feedback and understand the benefits to providers. In the event that providers habituate to messages at higher-than-anticipated rates during pilot implementation (Aim 2), we will develop and test requirements for message novelty. By using restricted randomization to minimize baseline imbalance [72], we will ensure that hospitals are allocated equitably and minimize the risk of bias. We will analyze the gender balance in participation and consider gender as a key factor in our process evaluation.

## Results

The proposal was funded in September 2021 with a 4-year timeline. Work on the technical integration of the precision feedback software with the MPOG email system began in January 2022. Data collection for Aim 1 began in March 2022, with 3 participants recruited at the time of manuscript submission. We plan for a 24-month trial timeline, with the intervention period of the trial beginning in March 2024.

## Discussion

### Hypothesis

Our primary hypothesis is that providers who receive precision A&F will increase care quality for improvable measures more than those who receive standard A&F emails. We also anticipate that engagement, in terms of email CTR and dashboard login rate, will be greater among providers receiving precision A&F.

### Comparison With Previous Work

The effects of A&F are mixed (median 4.3% absolute improvement, IQR 0.5%-16%) [7], indicating great uncertainty regarding how and when A&F works. Although this lack of knowledge has persisted for decades [5,74], A&F is increasingly being implemented electronically at a larger scale in clinical quality dashboards [2,3]. Furthermore, ongoing efforts to standardize and automate care quality measurement [1] further increase the potential volume of data for A&F. Without gaining knowledge about how and when A&F is effective, this inefficiency and the lost opportunities to improve care are likely to increase.

Best practice guidance about designing A&F offers methods for satisfying *most* providers’ requirements and preferences in a population to produce A&F that is usable and useful [48,75,76]. However, as an intervention scales up, its usability becomes increasingly important owing to the greater cost in time and attention across more diverse contexts [47]. Improving the usability of A&F requires recognition of multiple dimensions of *fit* for A&F, including the formatting of the message, the success of which depends on the characteristics of the message recipient, their context, and the visual representation itself [21,50,77,78]. Efforts to recognize this diversity have motivated our work to develop and study precision feedback interventions, which hold significant promise for improving the impact of digital interventions [12-15].

Understandably, efforts to improve A&F have focused on delivering actionable information to providers, which may be an important effect modifier of feedback interventions [6,29]. However, this focus can translate to the prioritization of negative feedback and social comparison that can demotivate providers [32,69,79]. For example, when a provider is learning a new skill, comparison with peers that show negative feedback can convince providers to abandon the skill-learning task [17,79]. Focusing solely on actionability results in a myopic view of the power of feedback interventions [80]. A broader view enables feedback interventions to leverage the motivation that arises from the recognition of achievements [31,33,69,81,82], even when performance is high, which can motivate increased effort, continuing learning, and goal setting [17].

CDS offers an extensive body of knowledge that could inform the study of A&F, exemplified by the CDS Five Rights framework [83] (right person, right information, right intervention format, right channel, and right timing in workflow); however, to our knowledge, this intersection of ideas remains largely unexplored [84]. A key issue that has prevented the application of CDS knowledge to A&F is a lack of well-defined terms and concepts used in A&F that hinder the ability of meta-analyses to recognize equivalent constructs for Five Rights. Efforts to standardize these elements [50,85] have yet to be broadly established. We developed an ontology of performance information in A&F reports, PSDO, and evaluated a sample of published reports [50], representing early progress toward this goal. Furthermore, a known problem with most CDS developed to date is that the causal mechanisms that CDS designers expect to drive improvement are not made explicit or even well understood [86]. Our approach to mass customization of A&F leverages causal mechanisms made explicit in our feedback intervention knowledge base that may benefit our understanding of its influence.

To our knowledge, this study will use for the first time an integrated representation of recipient requirements and preferences using theoretical constructs that direct the production of precision A&F messages. Using causal pathway models [53] (Figure 4) to model requirements and preferences as preconditions and moderators that affect intervention success, we gain the capability to prioritize messages based on theoretical mechanisms [17-19,29,32,87] and visual communication factors [21,27,77,78,88] together with what is known about the message recipient.

The proposed aims will collectively demonstrate a precision feedback service developed using an open-source technical infrastructure for computable knowledge management. We envision this approach to conform to the NIH National Library of Medicine’s vision of *data to knowledge* by demonstrating precision feedback using a *digital objects approach* [89,90]. By implementing and evaluating a demonstration system for precision feedback, we create the potential to observe the conditions under which feedback interventions are effective.

## Appendix

Multimedia Appendix 1 Peer-review report by the Biomedical Informatics, Library and Data Sciences Review Committee (National Institutes of Health, USA).

## Abbreviations

A&F: audit and feedback
API: application programming interface
CDS: clinical decision support
CTR: click-through rate
FAIR: Finable, Accessible, Interoperable, and Reusable
MPOG: Multicenter Perioperative Outcomes Group
NIH: National Institutes of Health
PSDO: Performance Summary Display Ontology
