# Supplementary material for: A Scalable Service to Improve Health Care Quality Through Precision Audit and Feedback: Proposal for a Randomized Controlled Trial
Source: JMIR Res Protoc. 2022 May 10;11(5):e34990. doi: 10.2196/34990 (PMC9131150; doi:10.2196/34990)
Supplement: Multimedia Appendix 1 [file resprot_v11i5e34990_app1.pdf]

1R01LM013894-01 Landis-Lewis, Zachary

EARLY STAGE INVESTIGATOR
NEW INVESTIGATOR

RESUME AND SUMMARYB OF DISCUSSION

Healthcare professionals often receive inadequate feedback about the quality of care they provide to their patients. The new principal investigator (PI) proposes to build a software service with “precision” audit and feedback (A&F), using anesthesia care as a demonstration domain. This is a well-written application, focused on improving the quality and safety of health care through tailored mechanisms for A&F, guided by formal theories, models, and frameworks. The approach is solid building on preliminary work, involving a national anesthesia quality improvement consortium with clearly described aims. The proposed approach constructs personalized and tailored emails each month to each provider, and it tracks improvements in quality as a primary outcome, compared against a one-size-fits-all email in the control arm. The PI provides information on potential problems, alternative approaches, and indicated benchmarks for success. The infrastructure is already in place, and there is much support for quality improvement initiatives in the setting where the project will take place. The investigators have extensive experience demonstrated through completed research projects and publications. The environment is outstanding. However, its single application domain (anesthesiology) may limit generalizability to other areas. Some additional details could further strengthen the approach. Overall, it is likely that the project will advance the creation of more general services for precision A&F in anesthesia care quality.

DESCRIPTION (provided by applicant):

All health care delivery organizations measure care quality and outcomes, increasingly via electronic clinical quality measures1 and dashboards2,3. However, these organizations lack evidence-based strategies for putting their quality and outcome data to work to improve performance4,5. The most common approach is audit and feedback (A&F), the delivery of clinical performance summaries to providers, which demonstrates potential for large effects on clinical practice6–8. But A&F too often produces negligible effects5,9, creating little more than distraction for providers who are fatigued by information chaos9–11. As currently implemented, A&F is a blunt, “one size fits most” intervention. Each provider in a care setting typically receives identical metrics in a common format, despite a growing recognition that “precisionizing” interventions holds significant promise to improve their impact12–15. A precision approach to A&F would prioritize display of information in the single metric that, for each recipient, carries the highest value for improving performance, such as when the metric's level drops below a peer benchmark or minimum standard for the first time, revealing an actionable performance gap16–19. Furthermore, precision A&F would employ an optimal message format (including framing and visual displays20–24), based on what is known about the recipient and the intended gist meaning being communicated, to improve message interpretation while reducing cognitive processing burden25–28. Well- established psychological principles, frameworks, and theoretical mechanisms provide a knowledge base to achieve precision A&F16–19,29–33. From an informatics perspective, precision A&F requires a knowledge-based system that uses psychological theory at its core, but which enables mass customization by giving precedence to configurable knowledge about recipients at the group and individual levels. A precision A&F service employs this knowledge as requirements (necessary characteristics for message acceptability) and preferences (the relative importance of message characteristics) to generate messages that are more likely than a “one size fits most” report to positively influence clinical decision-making and practice. An equally important informatics challenge is to enable widespread improvement through a service for precision A&F at scale. A scalable precision A&F service must function as infrastructure compatible with a wide range of computing environments and supporting a wide range of clinical domains. In his previous NLM K-award, the principal investigator developed and tested a prototype knowledge-based system for

precision A&F in email messages in anesthesia care. Preliminary data show that provider preferences are not uniform, suggesting that a platform for computable knowledge is necessary to support scalable precision A&F. The Knowledge Grid platform, developed at the University of Michigan, has been shown to support “precisionizing” for clinical decision support systems34–36. Based on our prior work, the proposed project will advance the creation of more general services for precision A&F, applying the service in anesthesia care as a demonstration domain.

PUBLIC HEALTH RELEVANCE:

Healthcare professionals often receive inadequate feedback about the quality of care they provide. We propose to build a software service that customizes feedback for healthcare professionals based on their requirements and preferences, and to evaluate the effect of using the service on care quality.

CRITIQUES:

The criterion scores provided below are given by individual reviewers assigned to this application and the critiques from reviewers are presented “as is”, without significant modification or editing by NLM staff. These individual critiques and criterion scores reflect the opinions of these assigned reviewers, which may or may not reflect the final Impact/Priority Score or final outcome/decision of the whole committee.

CRITIQUE 1

Significance: 3
Investigator(s): 1
Innovation: 4
Approach: 4
Environment: 1

Overall Impact: This R01 proposal from an ESI and NI from a K-award recipient seeks to develop a quality improvement system with “precision” audit & feedback, using anesthesia care as a demonstration domain. Aim 1 systematically captures user preferences and requirements. Aim 2 implements and assesses a demonstration system. Aim 3 assess the impact of the system on quality and intervention engagement. Studies are conducted in collaboration with the Multicenter Perioperative Outcomes Group (MPOG) which collects data on anesthesia quality from 50 hospitals and 5,000 providers. The proposed approach constructs personalized and tailored emails each month to each provider, and it tracks improvements in quality as a primary outcome, compared against a one-size-fits-all email in the control arm. Though this study is well designed, the limitation to a single application domain limits enthusiasm.

1. Significance:
Strengths

- Identifies and seeks to correct an important problem in medical quality improvement, and advances here could have a large impact on standardizing and improve care.
- Will be put into use at a national quality improvement consortium with about 5,000 different providers.
- Principled design of quality improvement emails to providers is an achievable goal that could improve quality of measurable quality indicators.

Weaknesses

- The inclusion of only a single demonstration domain limits generalizability to other areas and leaves open large questions about the difficult of developing this system for other domains.

- In this domain, performance is already high, which may limit impact if only focused on anesthesiology.

2. Investigator(s):
Strengths
- PI has strong training and experience in this specific area, with a PhD in bioinformatics and a successful K-award.
- Excellent inclusion of physicians in application domain

Weaknesses
- None noted.

3. Innovation:
Strengths
- Designs a “precision” decision support system using a knowledge base to emphasize feedback with (1) “highest value in increasing performance” and (2) customized to the healthcare provider preferences.

Weaknesses
- Customizing to health care provider may be counterproductive in some cases, shielding providers from information they should see but elect not to see.
- The proposal claims to be “the first comprehensive study of a novel approach to A&F” but Table 1 states that there is “Medium” precedence, without many references. So it is difficult to assess what precisely is the innovation here.

4. Approach:
Strengths
- Theory driven and guided approach, building off open source ontologies.
- Aim 1. Study design to elicit requirements and preferences among providers is well conceived and designed.
- Good consideration of research questions in Aim 3.
- Preliminary data mitigates risk, as does partnership with MPOG.

Weaknesses
- With only one application domain covered generalizability to other clinical domains is unstudied and unclear. Whether successful or not, it will be unclear what factors of the domain determine applicability. Proposal would be substantially improved by inclusion of an additional domain, at least in Aim 1, to assess generalizability of approach.
- How would this study determine cases where the user preference undermine the effectiveness of the system?
- No effort to consider how customization by health care providers might be counterproductive in some cases, shielding providers from information they should see but elect not to see.

5. Environment:
Strengths
- Excellent collaboration with MPOG.

Weaknesses
- None noted.

Protections for Human Subjects: Acceptable Risks and/or Adequate Protections - This falls into a grey-zone, and may not be a clinical study, because it is only provider behavior being study and reported in a non-identified way. It is not very different than an email marketing campaign, which would not typically even engage with an IRB.

Vertebrate Animals: Not Applicable

Biohazards: Not Applicable

Applications from Foreign Organizations: Not Applicable

Select Agents: Not Applicable

Resource Sharing Plans: Acceptable

Authentication of Key Biological and/or Chemical Resources: Not Applicable

Budget and Period of Support: No issues raised.

CRITIQUE 2

Significance: 2
Investigator(s): 2
Innovation: 2
Approach: 3
Environment: 2

Overall Impact: This is a well written R01 application from an early stage new investigator focused on improving the quality and safety of health care through tailored mechanisms for audit and feedback (A&F). The proposed development of a precision A&F service is guided by formal theories, models, and frameworks. The approach is solid building on preliminary work, involving a national anesthesia quality improvement consortium, and involving clearly described aims. The research team and environment are also strong.

1. Significance:
Strengths
- There is focus on improving quality and safety of health care through improved and tailored mechanisms for audit and feedback (A&F). For example, through clinical quality dashboards.

Weaknesses
- None noted.

2. Investigator(s):
Strengths
- PI is an early stage new investigator and prior recipient of an NLM K01 award who provides expertise in biomedical informatics and implementation science.
- Co-Is provide complementary expertise in informatics (Flynn) and anesthesiology quality improvement (Janda and Shah).

Weaknesses
- None noted

3. Innovation:
Strengths
- Develop precision A&F service for health care providers guided by formal theories, models, and frameworks (e.g., information value chain theory and causal pathway models)

Weaknesses
- None noted.

4. Approach:
Strengths
- Preliminary studies involving development and evaluation of relevant ontologies, knowledge bases, and message tailoring systems
- Involvement of a national anesthesia quality improvement consortium with >50 hospitals and >5,000 providers
- Clear aims focused on assessing requirements and preferences, implementing the precision A&F service, and studying the effects on the service on engagement and care.
- Sufficient description provided for methods, evaluation, potential challenges, etc.

Weaknesses
- Some additional details could further strengthen the approach (e.g., for the live usability testing and number of stakeholders involved in qualitative interviews).

5. Environment:
Strengths
- Excellent clinical and computing resources at the University of Michigan to support the proposed work

Weaknesses
- None noted

Protections for Human Subjects: Acceptable Risks and/or Adequate Protections
- Plans described for protecting data for human subjects (healthcare professionals).

Data and Safety Monitoring Plan: Not Applicable

Inclusion Plans:
- Sex/Gender: Distribution justified scientifically
- Race/Ethnicity: Distribution justified scientifically
- For NIH-Defined Phase III trials, Plans for valid design and analysis: Not applicable
- Inclusion/Exclusion Based on Age: Distribution justified scientifically
- No exclusions based on sex/gender and race/ethnicity. Adults only (healthcare professionals).

Vertebrate Animals: Not Applicable

Biohazards: Not Applicable

Applications from Foreign Organizations: Not Applicable

Select Agents: Not Applicable

Resource Sharing Plans: Acceptable - Described sharing of software, datasets, and other scientific products.

Authentication of Key Biological and/or Chemical Resources: Not Applicable

Budget and Period of Support: Recommend as Requested

CRITIQUE 3

Significance: 3
Investigator(s): 1

Innovation: 2
Approach: 2
Environment: 1

Overall Impact: Investigators aim to demonstrate the utility of mass customization of audit and feedback to improve care quality at large scale. Based on their prior work, the proposed project will advance the creation of more general services for precision A&F, applying the service in anesthesia care. Investigators have extensive experience demonstrated through completed research projects and publications. The proposed project is the next step towards precision A&F. The infrastructure is already in place, and there is much support for quality improvement initiatives in the setting where the project will take place. Going from one size fits most to precision A&F. The plan is methodical and well-thought out, and they have considered potential problems, alternative approaches, and indicated benchmarks for success.

1. Significance:
Strengths
- A precision approach to A&F would prioritize display of information for the single metric that, for each recipient, carries the highest value for improving performance. Well-established psychological principles, frameworks, and theories form a knowledge base to achieve precision.
- The significance of the proposed work derives from the widespread, almost universal--but largely ineffective--current use of audit and feedback (A&F) as a strategy to improve the quality and safety of health care. Improving the usability of A&F requires recognition of multiple dimensions of “fit” for A&F, including the formatting of the message, the success of which depends on characteristics of the message recipient, their context, and the visual representation itself. Efforts to overcome this diversity has motivated precision interventions, which hold significant promise to improve their impact.
- Instead of prioritizing negative feedback, which can demotivate care providers, the proposed A&F will focus on positive messages to recognize achievement and progress.
- Investigators will incorporate UX techniques, adaptive conjoint analysis to develop group and individual level requirements and preferences.
- Investigators propose to incorporate body of knowledge in the clinical decision support (CDS) framework (CDS Five Rights) into A&F. This has been difficult because of a lack of standardized terms in A&F that may have equivalent constructs in the CDS Five Rights. Investigators have already developed an ontology of visualized performance information in A&F reports that can be applied.
- Application of behavior change theories to feedback interventions

Weaknesses
- Provider performance is high, which could blunt the impact of precision feedback on performance.

2. Investigator(s):
Strengths
- PI and other key personnel have significant experience in the methods of knowledge representation, user-centered design focusing on audit and feedback to for healthcare quality improvement. They are affiliated with the Department of Learning Health Sciences and the Multicenter Perioperative Outcomes Group (MPOG) where they will be conducting their research. Landis-Lewis has developed a method for using feedback theory to specify the information content and form of feedback interventions. This work has yielded collaborations with feedback researchers in research network called the Audit and Feedback Metalab.
- Flynn is an information scientist and health informatics researcher who is the research lead for the Knowledge Grid program and its technical software development team. He also contributes to work focusing on defining and developing metadata for describing computable biomedical

knowledge (CBK) artifacts, including those needed for knowledge-based precision audit and feedback system and has 10 years prior experience working with EHR and clinical decision support systems. He also has completed related research in knowledge object and API development, as well as employing the USPSTF's Recommendations in computable form to achieve individualized precision prevention through scalable infrastructure, the Knowledge Grid. Janda is a physician researcher in cardiac anesthesiology and is affiliated with the MPOG and works on quality improvement issues for anesthesiology. She has experience with large-scale multicenter pragmatic clinical trials in anesthesia techniques. Shah has a background in clinical anesthesiology and experience building and implementing healthcare information technology to improve the workflow for clinicians. Shah has a unique background in EHR implementation, healthcare focused software development, and perioperative quality improvement. For the last 4 years, he has served as Program Director for the Anesthesiology Performance Improvement and Reporting Exchange, a quality improvement collaborative of 26 hospitals that leads efforts to improve anesthesia care in the state of Michigan. Shah is also the quality improvement director of the Multicenter Perioperative Outcomes Group (MPOG). Preliminary studies have already been conducted in knowledge representation, user-centered design, software development, and preference elicitation. These studies were conducted as part of 1) an NLM K01 award to develop a knowledge-based message tailoring system, 2) an A&F-based VA QUERI grant, and 3) a small grant from the University of Michigan Office of Research. Principal and Co-Investigators have significant publications that have contributed to the science relevant to this proposed project.

Weaknesses

- None noted by the reviewer

3. Innovation:

Strengths

- The most significant innovation is the ability to provide precision audit and feedback at scale, going from one size fits most to messages customized for each clinician. According to the investigators:
- This is the first comprehensive study of a novel approach to A&F for health care providers, in which each provider's individual performance data is analyzed across metrics to prioritize information that is most actionable and motivating.
- This study will employ for the first time an integrated representation of recipient requirements and preferences with theoretical constructs that direct the production of precision audit and feedback messages.
- The project proposes a novel approach, employing 3 customization strategies simultaneously, based on knowledge availability: a) theory-based customization using the characteristics of an individual's performance data, b) group-level segmentation and targeting based on user-centered design and preference data cluster analyses, and c) full tailoring using individual-level requirements and preferences obtained through user configuration of requirements and participation in a conjoint analysis survey.
- A scalable knowledge infrastructure for a precision A&F service: The proposed aims will collectively demonstrate a precision feedback service developed using an open-source technical computable biomedical knowledge management infrastructure. This infrastructure enables large-scale dissemination and management of knowledge that supports mass customization of audit and feedback. By implementing systems for deploying computable knowledge at large scale, the project team aims to create the potential to observe system-level learning about the conditions under which feedback interventions are effective at the individual, group, and universal level.

Weaknesses

- None noted by the reviewer

4. Approach:
Strengths

- Aim 1: Systematically capture recipient requirements and preferences for precision A&F messages
- Aim 2: Software service development and implementation study. Investigators will add an individualized message to the existing “one size fits most” A&F email sent monthly to 5,000+ providers and will evaluate the precision A&F service’s performance using existing quality measurement data from 50+ hospitals and conduct usability testing with a diverse sample of providers and hospitals.
- Aim 3: Investigators will conduct an embedded, pragmatic cluster-randomized trial of precision A&F-enhanced email vs a standard “one size fits most” A&F email to anesthesia providers.
- Project extends previous research investigator completed in A&F messaging.
- Study is theory based. The investigators addressed potential issues with bias and offered ways to prevent them.
- Primary and secondary outcomes are reasonable and measurable for Aim 3 trial and mixed methods process evaluation.
- Much preliminary work was done in prior research project.
- Although the first year is for developing requirements and preferences, implementing the service can begin the first year.
- The study will extend the MPOG infrastructure to use existing data and performance measurement processes

Weaknesses

- None noted by the reviewer

5. Environment:
Strengths

- The University of Michigan has established academic programs in two areas relevant to this application: health informatics and learning health system infrastructure. These academic programs are complemented and supported by a comprehensive library system that leads in developing the digital libraries of the future. Besides, advanced computational and networking resources are available at Michigan, including all of the IT design, development, implementation, and evaluation resources needed for this proposal. Michigan Medicine uses an advanced Electronic Health Record (EHR) system in all of its hospitals and clinics. It has an outstanding record of developing and implementing Clinical Decision Support (CDS) solutions in many biomedical domains.
- Multicenter Perioperative Outcomes Group (MPOG). Based at the University of Michigan, MPOG maintains a quality improvement infrastructure that represents a large-scale platform for research in precision feedback. Currently monthly A&F emails reach approximately 5,000 anesthesia providers.
- The University of Michigan, its Medical School, including the Departments of Anesthesiology and Learning Health Sciences, have a significant history of advancing learning health system, health IT, and digital health infrastructure research.
- Investigators also provided letters of support from the MPOG researcher director, a participant in the MPOG quality improvement provider feedback program, and a professor in the Department of Learning Health Sciences who will provide guidance to the research team on the use of data standards and metadata for digital objects.

Weaknesses

- None noted by the reviewer

Protections for Human Subjects: Acceptable Risks and/or Adequate Protections -Protections Include:

- Aim 1 and Aim 2 studies: A standardized process of informed consent will be used.
- Aim 3 trial: All healthcare professional participants will be notified about the study in advance by email and will be offered the option not to participate. They expect that few participants will "opt-out" because the intervention is a modification of an existing routine email that MPOG sends out to all participants in its monthly provider email feedback program.
- For all healthcare professional participants that recruited, a concise study overview packet for the particular study in which they agree to be involved will be shared. This is to ensure that (a) participants understand their rights and responsibilities, (b) the purpose of the study is clear to all participants, (c) the expectations of participants are clear, and (d) that participants are offered and made fully aware of the multiple public and private communication channels that are available for them to express any concerns about the study to the research team.
- Investigators will remove any identifiable information from recorded interview transcripts prior to analysis. All aggregate care quality data used in the proposed research is routinely collected and sent via email to all healthcare professionals in the population from which they will recruit, and this data will be kept securely under password protection.
- Minimum necessary data collection
- Study team training
- IRB oversight
- Data breach reporting

Data and Safety Monitoring Plan: Not Applicable

Inclusion Plans:
- Sex/Gender: Distribution justified scientifically
- Race/Ethnicity: Distribution justified scientifically
- For NIH-Defined Phase III trials, Plans for valid design and analysis: Not applicable
- Inclusion/Exclusion Based on Age: No age inclusion/exclusion indicated

Vertebrate Animals: Not Applicable

Biohazards: Not Applicable

Applications from Foreign Organizations: Not Applicable

Select Agents: Not Applicable

Resource Sharing Plans: Acceptable - PIs plan to share and disseminate the following research products:
- Software tools with user documentation
- Data sets collected from surveys
- Data sets collected from the generation of precision feedback messages
- Data sets collected from interviews
- Scientific reports, presentation materials, and peer-reviewed conference and journal articles
- They will use Open Software Code and Documentation Repositories (e.g., GitHub).

Authentication of Key Biological and/or Chemical Resources: Not Applicable

Budget and Period of Support: Recommend as Requested

THE FOLLOWING SECTIONS WERE PREPARED BY THE SCIENTIFIC REVIEW OFFICER TO SUMMARIZE THE OUTCOME OF DISCUSSIONS OF THE REVIEW COMMITTEE, OR REVIEWERS' WRITTEN CRITIQUES, ON THE FOLLOWING ISSUES:

PROTECTION OF HUMAN SUBJECTS: ACCEPTABLE

INCLUSION OF WOMEN PLAN: ACCEPTABLE

INCLUSION OF MINORITIES PLAN: ACCEPTABLE

INCLUSION ACROSS THE LIFESPAN PLAN: ACCEPTABLE

VERTEBRATE ANIMAL: NOT APPLICBLE

BIOHAZARDS: ACCEPTABLE/UNACCEPTABLE/NOT APPLICABLE

APPLICATIONS FROM FOREIGN ORGANIZATIONS: NOT APPLICABLE

SELECT AGENTS: NOT APPLICABLE

RESOURCE SHARING PLANS: ACCEPTABLE/UNACCEPTABLE/NOT APPLICABLE

AUTHENTICATION OF KEY BIOLOGICAL AND/OR CHEMICAL RESOURCES: NOT APPLICABLE

COMMITTEE BUDGET RECOMMENDATIONS: The budget was recommended as requested.

Footnotes for 1 R01 LM013894-01; PI Name: Landis-Lewis, Zachary

Ad hoc or special section application percentiled against "Total CSR" base.

NIH has modified its policy regarding the receipt of resubmissions (amended applications).See Guide Notice NOT-OD-18-197 at https://grants.nih.gov/grants/guide/notice-files/NOT-OD-18-197.html. The impact/priority score is calculated after discussion of an application by averaging the overall scores (1-9) given by all voting reviewers on the committee and multiplying by 10. The criterion scores are submitted prior to the meeting by the individual reviewers assigned to an application, and are not discussed specifically at the review meeting or calculated into the overall impact score. Some applications also receive a percentile ranking. For details on the review process, see http://grants.nih.gov/grants/peer_review_process.htm#scoring.
